# The Lesser Degrees of Chronic Pelvic Inflammations in Women

**Published:** 1884-10

**Authors:** 


### The Lesser Degrees of Chronic Pelvic Inflammations in Women.

Under this title there appears in the *New York Medical Journal* a most excellent paper from the pen of Dr. Frank P. Foster. Its practicability merits for it the careful perusal and thought of not only the gynæcologist, but of the general practitioner. He believes that the disorders of the generative apparatus and its functions, in a large proportion of cases, are due to a lesser degree of exudation in the peri-uterine cellular tissues, "in which no history of an acute beginning can be elicited, or any sign that there has been a tendency to suppuration, and in which there is no bulky exudate." "They are exceedingly common in occurrence; they are apt to be followed by consequences which produce serious impairment of health, and they constitute an important element to be considered, when we have to decide upon measures of treatment." Furtheron in his discourse, he says: "It is my decided conviction that this feature of these affections is underrated by many gynæcological writers. * * * Chapter after chapter is devoted to displacements of the uterus, to flexions of that organ, to dysmenorrhœa, to endometritis, and even to sterility, while the affections now under consideration—in my opinion, the most common beginning of them all—are treated as of minor consequence. * * * * * This one element seems to me ample to account for the great majority of cases of dysmenorrhœa, sterility, ovarian pain, profuse menstruation and leucorrhœa, that make up so large a share of the every-day practice of gynæcology." He then proceeds to show how injurious are many of the therapentic measures resorted to, in these cases, by those whose knowledge of the pathology and diagnosis of pelvic diseases is very limited. He mentions simply the introduction of the uterine sound and the possible restoration to its normal position of a displaced uterus. When it is considered how readily this chronic inflammation can be lighted into an acute inflammation, the reason of the possible injury is apparent. We commend the whole article to the careful perusal of all who have access to the *New York Medical Journal* of July 5, 1884.

